# Carcinome neuroendocrine thymique: à propos d’un cas et revue de la littérature

**DOI:** 10.11604/pamj.2017.26.25.11500

**Published:** 2017-01-18

**Authors:** Andriatsihoarana Voahary Nasandratriniavo Ramahandrisoa, Nomeharisoa Rodrigue Emile Hasiniatsy, Valéry Refeno, Clairette Raharisolo Vololonantenaina, Andriamihaja Jean Claude Rakotoarisoa, Hanitrala Jean Louis Rakotovao, Florine Rafaramino

**Affiliations:** 1 Faculté de Médecine d’Antananarivo, Madagascar; 2 Unité d’Oncologie Médicale, Centre Hospitalier de Soavinandriana, Madagascar; 3 Unité d’Anatomie Pathologique, Institut Pasteur de Madagascar; 4 Service Chirurgie Thoracique, Centre Hospitalier Universitaire Joseph Ravoahangy Andrianavalona, Madagascar

**Keywords:** Immunohistochimie, Madagascar, thymus, tumeurs neuroendocrines, Immunohistochemistry, Madagascar, thymus, neuroendocrine tumors

## Abstract

Les tumeurs neuroendocrines thymiques (TNET) sont rares, de pronostic mal connu. L'objectif est de rapporter un cas de TNET et discuter la difficulté diagnostique et thérapeutique en milieu à faibles ressources. Un homme de 60 ans présentait une douleur thoracique, une toux grasse et un amaigrissement récent. Le scanner thoracique révélait une masse tissulaire médiastinale antérieure. L'histologie définitive obtenue quatre mois après la biopsie par médiastinotomie antérieure montrait une TNET bien différenciée, avec marquage intense à la chromogranine et à la synaptophysine. La recherche d'autres tumeurs neuroendocrines et le bilan d'extension étaient négatifs. La tumeur était inextirpable d'emblée et la radiothérapie indisponible. Le patient a reçu deux lignes de chimiothérapie première. Avec un recul de 16 mois, le patient était asymptomatique, mais en progression tumorale. Le diagnostic de TNET peut être retardé quand l'examen immunohistochimique n'est pas réalisé en routine. La chimiothérapie apporte une amélioration des symptômes en situation palliative.

## Abstract (en)

Thymic neuroendocrine tumors (TNET) are rare, with little-known prognosis. This study aims to report a case of TNET and to highlight the diagnostic and therapeutic difficulties in low-resource settings. A 60-year-old man presented with chest pain, greasy cough and recent weight loss. Chest CT scan showed anterior mediastinal tissue mass. Histologic evaluation of a 4 months-biopsy specimen obtained from anterior mediastinotomy showed a well differentiated TNET, labeled intensely positive for chromogranin and synaptophysin. The search for other neuroendocrine tumors and the extent of extension were negative. The tumor was immediately inextricable and radiation therapy was unavailable. The patient underwent two lines of first line chemotherapy. At 16 months follow-up, the patient was asymptomatic but showed tumor progression. The diagnosis of TNET may be delayed when immunohistochemistry is not routinely performed. Chemotherapy is associated with symptoms improvement in palliative care situation.

## Introduction

Les tumeurs neuroendocrines thymiques (TNET) sont des tumeurs rares décrites pour la première fois en 1972 par Rosai et Higa sous le terme de tumeur carcinoïde thymique [1]. Le terme TNET a été introduit par Moran et Suster dans leur description de 80 cas au début des années 2000 [2]. Ce sont des tumeurs rares représentant environ 0,4 à 2 % de l'ensemble des tumeurs neuroendocrines et 2 à 4 % des tumeurs du médiastin antérieur [2–4]. De nombreux cas et petites séries ont été publiés partout dans le monde. Tout récemment, un cas nigérien de TNET a été décrit par Gaude [5]. A notre connaissance, aucun cas de TNET n'a été décrit à Madagascar. Ainsi nous avons comme objectifs de rapporter un cas de tumeur neuroendocrine thymique Malgache. Nous allons discuter la difficulté diagnostique dans un pays où l'examen immunohistochimique n'est pas utilisé en routine. Nous discutons également de la limite du traitement médical dans un pays où la radiothérapie est en panne pour une tumeur dont le stade chirurgical est dépassé.

## Patient et observation

**Figure 1:**
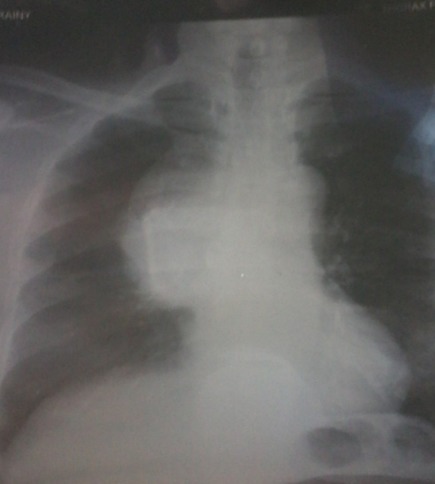
Radiographie du thorax: élargissement du médiastin, IMT 0, 4

**Figure 2:**
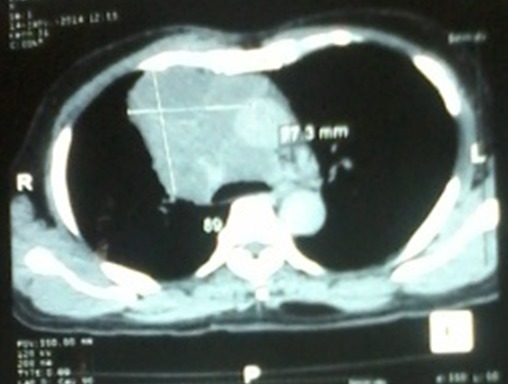
Scanner Thoracique: énorme masse médiastinale de 97, 3mm x89 mm

C'est le cas d'un homme d'origine malgache de 60 ans hypertendu grade III négligé, tabagique, avec une notion de tuberculose traitée et déclarée guérie. Il n'a ni antécédents personnels ni familiaux de cancer. Il a été adressé dans le Service de Pneumologie de l'Hôpital Militaire d'Antananarivo pour exploration d'une toux grasse productive récidivante évoluant depuis un an, associée à une douleur thoracique depuis six mois et un amaigrissement inexpliqué de 5 kg en 3 mois. L'état général a été conservé. Il rapportait une dysphonie, sans dyspnée ni hémoptysie. L'examen clinique initial était pauvre. Le test à la prostigmine était négatif. Il y avait un élargissement du médiastin avec un index médiastino-thoracique à 0,4 sur la radiographie du thorax (Figure 1). Le scanner montrait une masse médiastinale antérieure de 97,3 x 89 mm atteignant la paroi thoracique et englobant les gros vaisseaux (Figure 2). Le dosage des anticorps anti récepteurs de l'acetyl choline et de l'anticorps anti muscle lisse était négatif. D'autres examens paracliniques allant du moins invasif vers le plus invasif avaient été demandés pour l'exploration de cette masse. Le patient a bénéficié d'un dosage des marqueurs tumoraux, d'une fibroscopie bronchique et enfin d'une biopsie par médiastinotomie antérieure.

**Figure 3:**
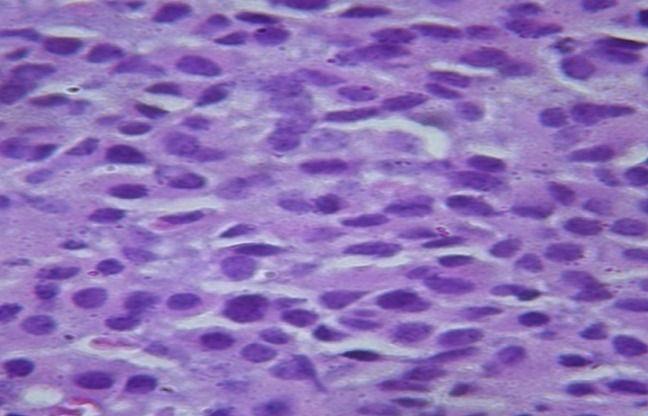
Coupe histologique montrant des cellules rondes à la coloration à l’hématine éosine au grossissement fois 40

Le dosage des marqueurs tumoraux notamment, la Lactate deshydrogenase (LDH), la sous unité beta de l'Hormone Chorionique Gonadotrope (ßHCG) et l'Antigène Carcino Embryonnaire (ACE) est revenu normal. La fibroscopie bronchique, montrait des cordes vocales symétriques et objectivait une compression extrinsèque des bronches. Le lavage bronchoalvéolaire était non contributif et la recherche de bacille acido alcoolo résistante (BAAR) était négative. Une biopsie par médiastinotomie antérieure avait été réalisée pour étayer le diagnostic. L'examen histologique revenait 12 jours plus tard et objectivait une prolifération faite de cellules rondes épithéliales, cohésives sans lymphocyte faisant évoquer initialement : un carcinome thymique, un lymphome B à grandes cellules, ou un thymome type B3 indiquant un examen immunohistochimique (Figure 3).

**Figure 4:**
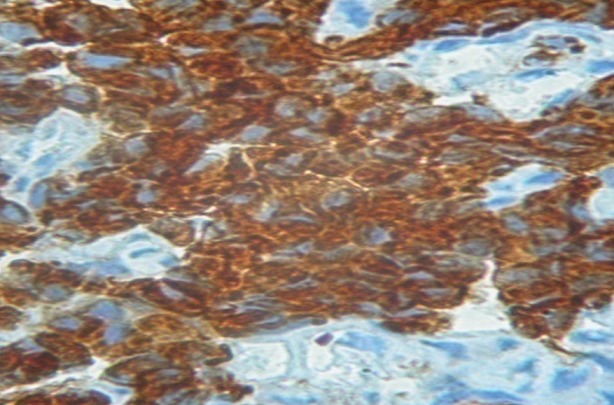
Immunohistochimie: cellules positives à la cytokératine AE. Grossissement fois 40

**Figure 5:**
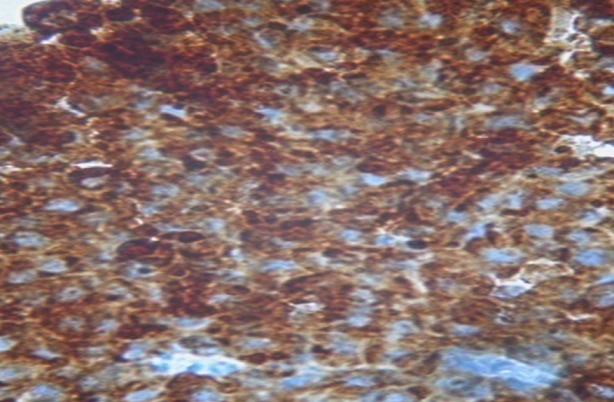
Immunohistochimie: cellules positives au chromogranine A. Grossissement fois 40

**Figure 6:**
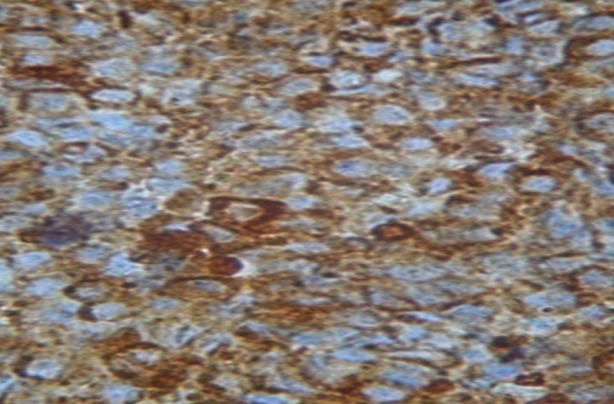
Immunohistochimie: cellules positives à la synaptophysine. Grossissement fois 40

L'immunohistochimie était demandée à la réception du résultat de l'examen morphologique, et revenait 43 jours après la biopsie. Elle montrait un marquage important à la cytokératine (Figure 4), à la chromogranine (Figure 5) et à la synaptophysine (Figure 6) posant le diagnostic de carcinome neuroendocrine thymique. Une relecture de la pièce demandée pour plus de précision revenait 3 mois plus tard (4 mois et demi après la biopsie), la tumeur était alors classée en tumeur bien différenciée. La recherche d'autres localisations neuroendocriniennes était négative (Tableau 1). Devant le caractère non résécable, l'absence de radiothérapie à Madagascar et l'impossibilité financière pour une évacuation sanitaire, une chimiothérapie de type cisplatine, étoposide avait été instituée. L'évaluation après 6 cycles montrait une progression tumorale, mais le patient était en bon état général et asymptomatique (disparition de la douleur thoracique et de la dysphonie). Une deuxième ligne de chimiothérapie type 5 fluoro uracile, dacarbazine, adriblastine était instituée. A 16 mois du diagnostic, la masse est en progression malgré les 6 cycles de chimiothérapie de deuxième ligne, mais son état général est toujours conservé. Il est en surveillance dans l'attente de l'ouverture prochaine de la radiothérapie à Madagascar.

**Tableau 1:** Le bilan réalisé à la recherche d’autres tumeurs neuroendocrines

Autres tumeurs neuroendocrines	Biologie	Imagerie
Hypophyse	ACTH: normal	Scanner cérébral normal
Thyroïde	Néant	Echographie thyroïdienne: normale
Parathyroïde	Parathormone: normale	Néant
Digestives	Insuline: normal Gastrine: normal Glucagon: normal Somatostatine: normal	Pas de syndrome de masse abdominale
Corticosurrénales	Cortisol: normal	Pas de lésions surrénaliennes

## Discussion

La plus grande série de TNET rapportant 80 cas a été rapportée par Moran et Suster en 2000 [2]. A notre connaissance, c'est le premier cas de TNET rapportée chez les malgaches. Ces tumeurs surviennent surtout chez l'homme, aux alentours de la sixième décennie avec un sex ratio de 3/1 [2–4, 6]. Notre cas rentre dans cette catégorie de patients.

Ces tumeurs sont asymptomatiques dans 30% des cas et découvertes fortuitement lors d'un examen d'imagerie de routine [2, 6, 7]. Des signes non spécifiques en rapport avec la compression des organes de voisinage (toux, dyspnée, douleur thoracique, syndrome cave supérieur…) peuvent s'observer [5, 6]. La toux chronique et la douleur thoracique étaient les circonstances révélatrices pour notre cas. L'amaigrissement a été également retrouvé par Moran et Suster dans 6% des cas [2]. La moitié de ces tumeurs seraient associées à des endocrinopathies [4, 6, 7]. Ce qui n'a pas été le cas pour notre patient. Le scanner thoracique montre typiquement une énorme masse lobulée et invasive, avec parfois des plages de nécroses et de calcifications [ 6, 7]. Bien que l'imagerie par résonance magnétique nucléaire est l'examen de choix pour la détection des atteintes des organes de voisinage permettant ainsi d'apprécier l'extension locorégionale de la maladie [6]. Cet examen n'est disponible que dans un centre de soins à Madagascar, notre patient n'en avait pas bénéficié. Le scanner thoraco-abdomino-pelvien a permis d'objectiver l'extension loco-régionale le caractère non résecable de la tumeur de notre patient. Dans 20 % des cas, la maladie peut se manifester par des métastases révélatrices [6, 7], notamment les métastases pleurales, pulmonaires et hépatiques. Des localisations à distance n'ont pas été retrouvées chez notre patient.

Les tumeurs neuro-endocrines thymiques sont décrites à l'histologie comme des tumeurs épithéliales, faites de cellules rondes à noyaux ronds ou ovalaires [4]. Dans notre cas, elles étaient cohésives, sans lymphocytes. L'aspect histologique des tumeurs neuroendocrines thymiques peuvent avoir une similarité avec les autres masses médiastinales notamment les thymomes, le lymphome, le paragangliome, le carcinome médullaire de la thyroïde etc [2, 4]. Pour notre cas, trois diagnostics ont été évoqués à l'histologie : carcinome thymique, lymphome B à grandes cellules et un thymome type B3.

L'examen immunohistochimique est indispensable pour démontrer la différenciation neuroendocrine de ces tumeurs. Elle ne se fait pas d'emblée mais est réalisée secondairement à la demande du clinicien, d'où le délai long pour avoir le diagnostic. Les marqueurs neuroendocrines généraux utilisés par Moran et Suster ainsi que Chalabreysse étaient la synaptophysine (SP), les chromogranines (Cg) A, B et C et enfin la N CAM (Neural Cell Adhesion Molecule) [2, 4]. La synaptophysine et la chromogranine A sont les marqueurs les plus fréquemment utilisés et l'expression des cytokératines confirme l'origine épithéliale de la tumeur [2, 5]. Dans notre cas le diagnostic de tumeur neuroendocrine thymique a été posé devant la positivité de la cytokératine AE1/AE3, la synaptophysine et la chromogranine A. La détermination de la différenciation tumorale a été réalisée après la confirmation immunohistochimique de la maladie. Le diagnostic définitif a été obtenu à plus de 4 mois et demi de la biopsie. Moran et Suster de l'AFIP (Armed Forces Institute of Pathology) distingue les tumeurs bien différenciées, moyennement différenciées et peu différenciées [2]. Les formes moyennement différenciées sont les plus rencontrées [2, 7], nous avons décrit une tumeur bien différenciée.

Le rôle pronostique de la différenciation tumorale est très discuté. En effet, les données de la littérature sont divergentes. Selon Moran, le pronostic est directement lié au degré de différenciation [2]. Pour Crona, un taux de prolifération tumorale basse est accompagné d'une meilleure survie [8]. Pour Filosso et al, selon les résultats d'une étude multicentrique menée par l'ITMIG (International Malignancy Interest Group) et l'European Society of Thoracic Surgeons (ESTS), le degré de différenciation n'influence pas la survie. Cette dernière serait plutôt corrélée au stade de la maladie et la qualité de la résection [7]. Pour Crona, la résection et la qualité macroscopique de cette résection sont des facteurs pronostiques déterminant la survie [8]. La classification en grade de European Neuroendocrine Tumor Society (ENETS) et le stade tumoral selon Masaoka ne sont pas corrélés à la survie [8]. Notre cas est stade III de Masaoka bien différencié.

Dans les quelques cas des séries antérieurement rapportées, une exérèse complète de la tumeur a été réalisée après le traitement d'induction [3, 7, 9]. Cardillo et al ont décrit le bénéfice du traitement chirurgical après chimiothérapie ou radiothérapie néoadjuvante. La survie médiane était de 153 mois [9]. Dans d'autres cas, une évolution défavorable a été rencontrée. Fukai et son équipe rapportait une série de 15 patients ayant tous bénéficié d'un traitement chirurgical. La résection était complète pour 13 patients, huit patients ont bénéficié d'une radiothérapie adjuvante. Parmi ces patients, dix développaient une récidive métastatique ; six décédaient dans les 5 à 24 mois après la récidive ; seul un patient survivait cinq ans après le diagnostic [3].

Dans tous les cas, la chirurgie reste le traitement standard de ce type de tumeur [1–4, 6, 8, 9]. La chirurgie rentre dans le cadre d'un traitement multimodal associant chirurgie, chimiothérapie et radiothérapie [3]. Notre patient n'a pas pu bénéficier de chirurgie à cause de son caractère non résécable d'emblée et l'absence de réponse tumorale objective après chimiothérapie néoadjuvante. Concernant la chimiothérapie, aucune molécule standard n'existe actuellement. Les plus fréquemment utilisées dans les quelques séries rapportées sont l'association Cisplatine, Etoposide, Dacarbazine, Vincristine, Cyclophosphamide, 5Fluoro uracile, Streptozocine, Carmustine [7, 9, 10]. D'autres drogues comme la Capécitabine, le Temozolomide et les thérapies ciblées comme le Sorafenib ou le Bevacizumab ont été utilisés tout récemment [8]. La combinaison de chimiothérapie que nous avons utilisé pour les deux lignes ont permis d'améliorer les symptômes initiaux. Selon les séries, la chimiothérapie et la radiothérapie sont utilisées en induction ou en adjuvant [3, 9]. La radiothérapie n'était pas disponible lors de la prise en charge de notre patient. Un cas africain localement avancé envahissant les gros vaisseaux la paroi thoracique décrit par Gaude et al est comparable au nôtre. Il était décédé cinq mois après le diagnostic malgré une chirurgie première incomplète, suivie d'une radiothérapie adjuvante et de 4 cycles de chimiothérapie type cisplatine, bléomycine et adriblastine [5]. Notre patient est vivant à 16 mois du diagnostic, son état général est conservé, il est asymptomatique malgré la progression tumorale iconographique. En attente de l'ouverture de la radiothérapie à Madagascar, il bénéficiera d'une pause thérapeutique avec surveillance clinique et iconographique.

## Conclusion

Les tumeurs neuroendocrines thymiques sont des tumeurs rares qui existent chez les Malgaches. L'immunohistochimie est décisive pour asseoir le diagnostic, son accès devrait être facilité à Madagascar. La prise en charge de ces tumeurs est difficile lorsque la chirurgie ne peut pas être effectuée. En l'absence de radiothérapie, la chimiothérapie palliative pourrait être une option pour améliorer la symptomatologie clinique.
